# The islet tissue plasminogen activator/plasmin system is upregulated with human islet amyloid polypeptide aggregation and protects beta cells from aggregation-induced toxicity

**DOI:** 10.1007/s00125-024-06161-0

**Published:** 2024-09-09

**Authors:** Nathalie Esser, Meghan F. Hogan, Andrew T. Templin, Rehana Akter, Brendy S. Fountaine, Joseph J. Castillo, Assam El-Osta, Lakshan Manathunga, Alexander Zhyvoloup, Daniel P. Raleigh, Sakeneh Zraika, Rebecca L. Hull, Steven E. Kahn

**Affiliations:** 1 Veterans Affairs Puget Sound Health Care System, Seattle, WA USA; 2 Division of Metabolism, Endocrinology and Nutrition, Department of Medicine, University of Washington, Seattle, WA USA; 3 Laboratory of Immunometabolism and Nutrition, GIGA, University of Liège, CHU of Liège, Liège, Belgium; 4 Division of Diabetes, Nutrition and Metabolic Disorders, Department of Medicine, CHU of Liège, Liège, Belgium; 5 Division of Endocrinology, Department of Medicine, Roudebush VA Medical Center and Indiana University School of Medicine, Indianapolis, IN USA; 6 Epigenetics in Human Health and Disease Program, Baker Heart and Diabetes Institute, Melbourne, VIC Australia; 7 Department of Chemistry, Stony Brook University, Stony Brook, NY USA; 8 Laufer Center for Physical and Quantitative Biology, Stony Brook University, Stony Brook, NY USA; 9 Research Department of Structural and Molecular Biology, University College London, London, UK

**Keywords:** Amyloid, Beta cell, Fibrinolysis, Human islet, Islet amyloid polypeptide, Plasmin, Tissue plasminogen activator, Type 2 diabetes

## Abstract

**Aims/hypothesis:**

Apart from its fibrinolytic activity, the tissue plasminogen activator (tPA)/plasmin system has been reported to cleave the peptide amyloid beta, attenuating brain amyloid deposition in Alzheimer’s disease. As aggregation of human islet amyloid polypeptide (hIAPP) is toxic to beta cells, we sought to determine whether activation of the fibrinolytic system can also reduce islet amyloid deposition and its cytotoxic effects, which are both observed in type 2 diabetes.

**Methods:**

The expression of *Plat* (encoding tPA) and plasmin activity were measured in isolated islets from amyloid-prone hIAPP transgenic mice or non-transgenic control islets expressing non-amyloidogenic mouse islet amyloid polypeptide cultured in the absence or presence of the amyloid inhibitor Congo Red. *Plat* expression was also determined in hIAPP-treated primary islet endothelial cells, bone marrow-derived macrophages (BMDM) and INS-1 cells, in order to determine the islet cell type(s) producing tPA in response to hIAPP aggregation. Cell-free thioflavin-T assays and MS were used to respectively monitor hIAPP aggregation kinetics and investigate plasmin cleavage of hIAPP. Cell viability was assessed in INS-1 beta cells treated with hIAPP with or without plasmin. Finally, to confirm the findings in human samples, *PLAT* expression was measured in freshly isolated islets from donors with and without type 2 diabetes.

**Results:**

In isolated islets from transgenic mice, islet *Plat* expression and plasmin activity increased significantly with the process of amyloid deposition (*p*≤0.01, *n*=5); these effects were not observed in islets from non-transgenic mice and were blocked by Congo Red (*p*≤0.01, *n*=4). In response to hIAPP exposure, *Plat* expression increased in BMDM and INS-1 cells vs vehicle-treated cells (*p*≤0.05, *n*=4), but not in islet endothelial cells. Plasmin reduced hIAPP fibril formation in a dose-dependent manner in a cell-free system, and restored hIAPP-induced loss of cell viability in INS-1 beta cells (*p*≤0.01, *n*=5). Plasmin cleaved monomeric hIAPP, inducing a rapid decrease in the abundance of full-length hIAPP and the appearance of hIAPP 1–11 and 12–37 fragments. hIAPP 12–37, which contains the critical amyloidogenic region, was not toxic to INS-1 cells. Finally, *PLAT* expression was significantly increased by 2.4-fold in islets from donors with type 2 diabetes (*n*=4) vs islets from donors without type 2 diabetes (*n*=7) (*p*≤0.05).

**Conclusions/interpretation:**

The fibrinolytic system is upregulated in islets with hIAPP aggregation. Plasmin rapidly degrades hIAPP, limiting its aggregation into amyloid and thus protecting beta cells from hIAPP-induced toxicity. Thus, increasing islet plasmin activity might be a strategy to limit beta cell loss in type 2 diabetes.

**Graphical Abstract:**

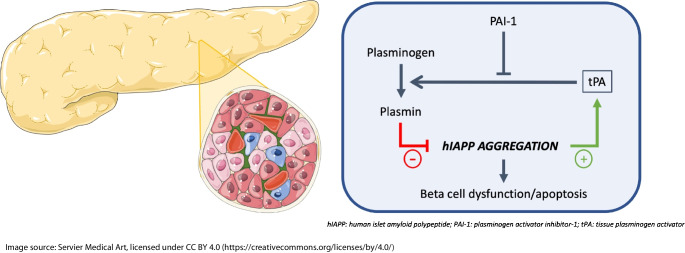

**Supplementary Information:**

The online version contains supplementary material available at 10.1007/s00125-024-06161-0.


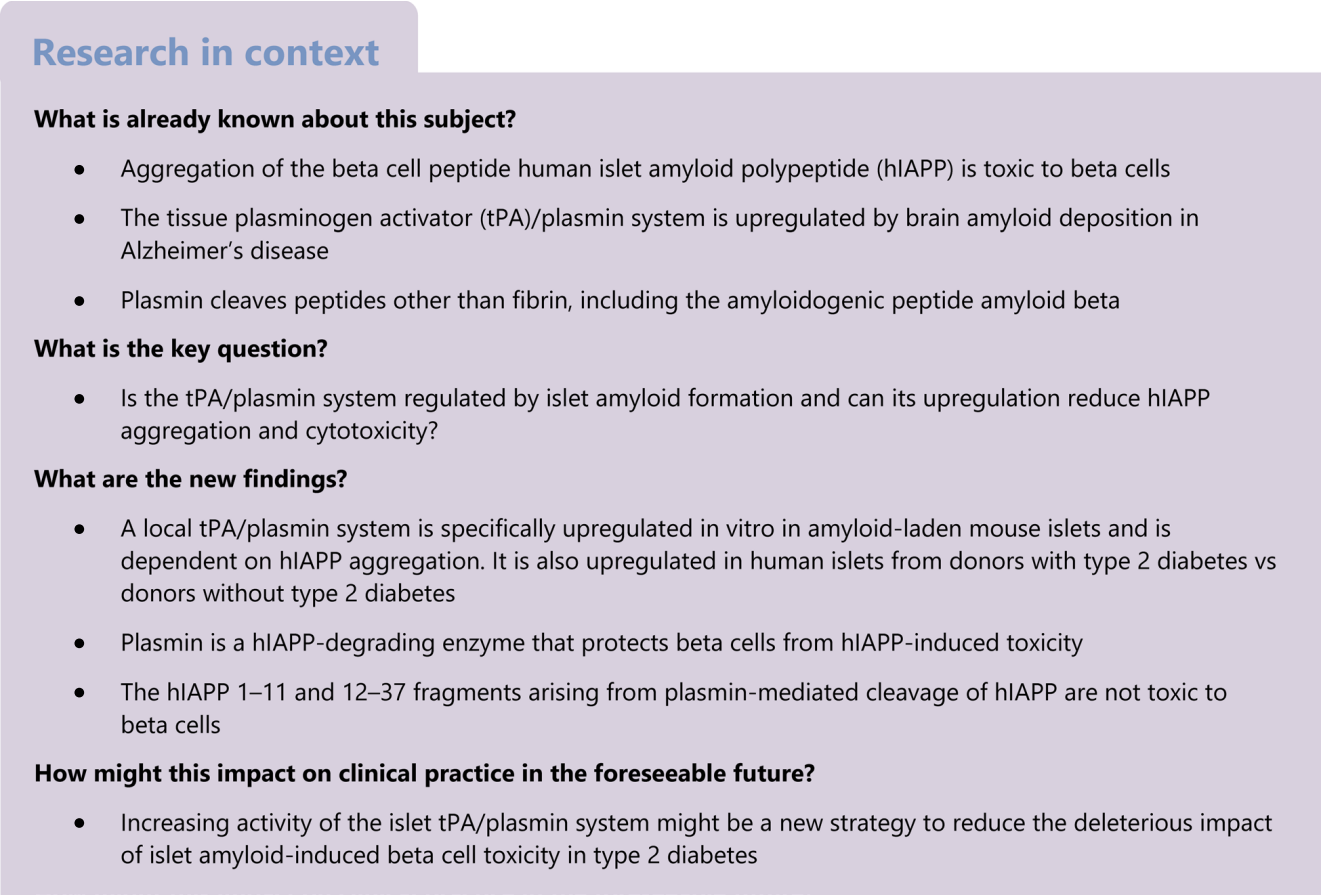


## Introduction

Type 2 diabetes is characterised by islet amyloid deposition, which is associated with beta cell loss and dysfunction [1–3]. Amyloid deposits contain the normal beta cell secretory product human islet amyloid polypeptide (hIAPP; also known as amylin) [4]. Since the process of hIAPP aggregation is toxic to beta cells [3, 5], the development of approaches to limit hIAPP aggregation could be beneficial for slowing or preventing beta cell loss in type 2 diabetes.

hIAPP is a 37 amino acid peptide, wherein the region comprising amino acids 20–29 plays an important role in dictating amyloidogenicity [6–8]. In contrast, due to several critical amino acid differences in this sequence and an H18R substitution, mouse islet amyloid polypeptide (mIAPP) is neither amyloidogenic nor cytotoxic [6]. Thus, transgenic mice expressing amyloidogenic hIAPP specifically in their beta cells have been produced to study islet amyloid formation. These mice develop amyloid deposits that are morphologically indistinguishable from those observed in human type 2 diabetes [9, 10] and have been used for both in vitro and in vivo studies of hIAPP aggregation and islet-amyloid-induced beta cell loss [9–12].

Based on bulk transcriptome analysis of amyloid-laden hIAPP mouse islets [13], we identified *Plat* as a gene specifically upregulated in islets under amyloid-forming conditions. *Plat* encodes tissue plasminogen activator (tPA), a secreted serine protease that initiates fibrinolysis by cleaving the circulating proenzyme plasminogen into the active protease plasmin, which, in turn, degrades fibrin blood clots [14]. In this process, fibrin is required for efficient tPA-mediated plasminogen activation [15]. Interestingly, both the amyloidogenic peptides hIAPP and amyloid beta (Aβ; the unique constituent of brain amyloid in Alzheimer’s disease) can bind to tPA and substitute for fibrin in the tPA activation of plasminogen [16, 17]. Since the tPA/plasmin system has been reported to attenuate brain amyloid deposition by cleaving Aβ [18–20], we hypothesised that it may also be effective in reducing islet amyloid deposition and its cytotoxic effects in type 2 diabetes. In this study, we examined: (1) whether the tPA/plasmin system is upregulated in vitro in amyloid-laden mouse islets and in islets isolated from donors with type 2 diabetes; (2) the islet cell types expressing tPA; and (3) whether plasmin can limit hIAPP aggregation in a cell-free system and reduce hIAPP-induced beta cell toxicity.

## Methods

### Isolation and culture of mouse islets

Transgenic mice with hemizygous expression of hIAPP under the rat insulin promotor (B6D2-Tg(RIP-hIAPP)CStka; generated as described in [21]) were bred on an F1 C57BL/6 × DBA/2J background [10, 12]. Non-transgenic littermates were used as controls. Mice were housed and bred in a specific-pathogen-free vivarium at VA Puget Sound Health Care System, with ad libitum access to food and water. All animal studies described below were approved by the Institutional Animal Care and Use Committee at VA Puget Sound Health Care System, performed in an AAALAC-accredited animal research facility, and adhered to the Animal Research: Reporting of In Vivo Experiments guidelines.

Islets from 8–12 week-old male and female mice were isolated by collagenase digestion, as previously described [12]. Islets were handpicked and cultured overnight in complete RPMI-1640 medium containing 10% (vol./vol.) FBS, 1 mmol/l sodium pyruvate, 100 U/ml penicillin, 100 μg/ml streptomycin and 11.1 mmol/l glucose (complete medium). Thereafter, islets were distributed via a block randomisation method into islet pools and cultured for up to 144 h in complete RPMI medium containing either 11.1 mmol/l or 16.7 mmol/l glucose, the latter to induce amyloid deposition in hIAPP islets. The culture medium was renewed every 48 h. Subsets of islets were cultured for 48 h in the presence of Congo Red (200 μmol/l) or its vehicle control (DMSO), as done previously [22]. At the end of each experimental culture period, islets were collected for RNA extraction, plasmin activity measurement and/or histology, as described below.

For islet macrophage depletion, freshly isolated islets were cultured for 48 h in complete RPMI medium with 1 mg/ml clodronate-containing liposomes (Liposoma, Amsterdam, the Netherlands), or 1 mg/ml PBS-containing liposomes (Liposoma) or PBS alone as controls. Thereafter, they were transferred into complete RPMI medium containing 16.7 mmol/l glucose and cultured for 48 h, with the goal of inducing amyloid deposition in hIAPP transgenic islets. Subsequently, islets were collected for RNA extraction.

### Islet plasmin activity assay

Plasmin enzymatic activity in islet protein lysates was assayed by measuring the release of para-nitroaniline from the chromogenic substrate of plasmin S-2251 (Molecular Innovation, MI, USA). Briefly, Glu-plasminogen (0.5 μmol/l in 50 mmol/l Tris-HCl+100 mmol/l NaCl, pH 7.4; Molecular Innovation) and then S-2251 (0.4 mmol/l) were added to islet lysate samples (10 μg protein/sample, in duplicate) and mixed. The subsequent colour change was quantified at 405 nm after 120 min of incubation at 37°C on a Beckman Coulter DTX880 plate reader (Beckman Coulter, CA, USA).

### Histology and quantitative microscopy

Islets were formalin-fixed, paraffin-embedded and sectioned (10 µm). Sections were labelled with anti-insulin antibody (1:2000; Sigma-Aldrich, USA; catalogue no. I2018; RRID:AB_260137), followed by Cy3-conjugated goat anti-mouse IgG (1:250; Jackson ImmunoResearch Labs, West Grove, PA, USA; catalogue no. 115-165-146; RRID:AB_2338690, USA) and counterstaining with thioflavin-S (0.5% [wt/vol.] in aqueous solution; Sigma-Aldrich) to visualise beta cells and amyloid deposits [12]. The anti-insulin antibody was selected based on extensive validation in house. Sections were blocked for 1 h in buffer containing 0.05 mol/l PBS, 0.2% (wt/vol.) Triton X-100 (Sigma-Aldrich), 0.01% (wt/vol.) sodium azide (Sigma-Aldrich), 1% (wt/vol.) BSA (Sigma-Aldrich) and 2% normal goat serum (Vector Laboratories, USA). Antibodies were diluted in buffer containing 0.05 mol/l PBS, 0.2% (wt/vol.) Triton X-100, 0.01% (wt/vol.) sodium azide and 1% (wt/vol.) BSA. Islet images were acquired and analysed using a custom semi-automated workflow (Nikon TiE wide field microscope and Nikon NIS Elements AR v5.02.01 software; Nikon, USA). Briefly, islets were identified based on insulin immunofluorescence from a large area (×2) scan, and multichannel images were acquired at ×20. From these images, thioflavin-S-positive areas were computed based on pre-set pixel-density thresholds, using an automated method based on our previous manual approach [12] and image post-processing to compute islet cross-sectional areas. Amyloid prevalence was defined as the number of amyloid-positive islets/total number of islets×100, and amyloid severity as amyloid area/islet area×100 [3, 9, 13]. A mean of 18.5±1.8 islets per condition were analysed. The observer was blinded to genotype and culture conditions.

### Peptide synthesis and purification

Full-length hIAPP, hIAPP 1–11 and hIAPP 12–37 were synthesised using 9-fluorenylmethylcarbonyl (Fmoc) chemistry on a 0.10 mmol scale with a CEM Liberty Blue peptide synthesiser (CEM, USA) [23]. Fmoc-PAL-PEG-PS resin (Agilent; 0.19 mmol/eq) was used for C-terminal amidation of full-length hIAPP and hIAPP 12–37. Peptides were cleaved from the resin using a trifluoroacetic acid (TFA)-based cleavage cocktail (92.5% [vol./vol.] TFA, 2.5% [vol./vol.] triisopropylsilane, 2.5% [vol./vol.] 3,6-dioxa-1,8-octanedithiol and 2.5% [vol./vol.] water). Crude peptides were dried and then dissolved in 20% acetic acid (4 mg/ml) followed by lyophilisation to improve their solubility. The Cys2 and Cys7 disulfide bridge was assembled by oxidising the crude peptide in 100% DMSO (10 mg/ml). Reverse-phase HPLC was used to purify the peptides. A Higgins Analytical C18 preparative column (Higgins Analytical, USA), 25 mm×250 mm, was employed with a binary A-B gradient of water and acetonitrile with 0.1% (vol./vol.) TFA. The purified peptides were lyophilised and redissolved in 1,1,1,3,3,3-hexafluoroisopropanol and subjected to a second round of reverse-phase HPLC purification. Analytical HPLC was used to confirm peptide purity and matrix-assisted laser desorption ionisation time-of-flight MS was used to verify the expected mass. mIAPP was purchased from Amyloid Peptide LLC (Danbury, CT, USA).

For cell treatment, islet amyloid polypeptide (IAPP) peptides were resuspended in Tris-HCl buffer (20 mmol/l, pH 7.4) to a final concentration of 250 μmol/l and then, immediately prior to use, diluted into complete media at final concentrations of 0–60 μmol/l.

### Thioflavin-T fluorescence assays

Kinetics of amyloid formation were determined using solutions containing hIAPP 1–37 and/or hIAPP 1–11 and/or hIAPP 12–37, and thioflavin-T (32 μmol/l; catalogue no. T3516; Sigma), and Tris-HCl (20 mmol/l, pH 7.4) in the presence or absence of plasmin (0–4 μmol/l; Molecular Innovation) or tPA (8 nmol/l; Molecular Innovation). Samples were incubated in triplicate at 25°C or 37°C, with plate shaking every 10 min, for up to 72 h. Fluorescence was recorded every 10 min on a Beckman Coulter DTX880 plate reader using an excitation wavelength of 450 nm and an emission wavelength of 485 nm. Control reactions were carried out in the absence of hIAPP peptides.

### Negative stain transmission electron microscopy

Samples (15 μl) of material collected at the end of the thioflavin-T assays were blotted onto carbon-coated formvar 300 mesh copper grids (Electron Microscopy Sciences, USA). The same volume of 1% (wt/vol.) depleted uranyl acetate was used to stain each sample. Images were recorded at the Central Microscopy Imaging Center facility at Stony Brook University (Stony Brook, NY, USA).

### MS

Full-length hIAPP (20 μmol/l) was incubated with or without human recombinant plasmin (0.4 μmol/l and 4 μmol/l; Molecular Innovation) at 37°C for up to 8 h. Samples were analysed by LC/MS after the indicated incubation time on an LTQ-Orbitrap XL mass spectrometer (ThermoFisher, USA).

### Beta cell line experiments

The beta cell line INS-1 832/13 (RRID: CVCL_7226), originally provided by C. Wollheim (University of Geneva, Geneva, Switzerland) [24], which was negative for mycoplasma, was cultured in complete RPMI medium. In total, 15×10^3^ cells/well were plated in triplicate into a gelatin-coated 96-well plate and incubated until confluency, after which the medium was replaced with complete RPMI medium containing freshly dissolved hIAPP peptides (0–60 μmol/l) or Tris-HCl buffer (20 mmol/l, pH 7.4), as a control. After a 24 h incubation, cell viability was assessed using the fluorescent CellTiter-Fluor viability assay (Promega, Madison, WI, USA). Absorbance of a blank sample (no cells) was used to determine assay background, which was subtracted from every experimental sample. Each sample was normalised to buffer-treated cells. In a subset of experiments, cells were collected for RNA extraction at the end of the 24 h incubation with hIAPP (20 μmol/l) or Tris-HCl buffer (20 mmol/l, pH 7.4), as control.

### Bone marrow-derived macrophage experiments

Femur marrow from 6 month-old male Sprague Dawley rats (Charles River Laboratories, USA; www.criver.com/products-services/find-model/cd-sd-igs-rat?region=3611) was differentiated for 6 days in RPMI+10% (vol./vol.) FBS and 25 ng/ml recombinant human macrophage colony-stimulating factor (M-CSF; ThermoFisher) to make bone marrow-derived macrophages (BMDM). BMDM were then lifted from tissue culture plates using ice-cold PBS with 2 mmol/l EDTA, washed, plated and cultured overnight in RPMI+10% (vol./vol.) FBS and 25 ng/ml M-CSF. Subsequently, the medium was replaced with RPMI+10% (vol./vol.) FBS. After 48 h, the medium was replaced with complete RPMI containing freshly dissolved IAPP peptide (10 μmol/l) or Tris-HCl buffer (20 mmol/l, pH 7.4), as control. BMDM were incubated for 24 h and then collected for RNA extraction.

### Human islets

Human islets from male and female donors with or without type 2 diabetes were obtained from the Integrated Islet Distribution Program. Characteristics of the donors are listed in the electronic supplementary material (ESM) Table 1 (human islets checklist). Sex of the donors was determined based on the medical history from the organ procurement organisation via the isolation centre. Freshly isolated shipped islets (25–50 per donor) were collected for RNA extraction. As the human islets were anonymised, their use was not considered human research by the institutional review board.

### Gene expression analysis

Total RNA was extracted from islets or cells using the High Pure RNA Isolation Kit (Roche, Basel, Switzerland), reverse-transcribed using the High-Capacity cDNA Reverse Transcription Kit (ThermoFisher) and then subjected to quantitative RT-PCR (qRT-PCR).

cDNA samples from primary islet endothelial cells isolated from Sprague Dawley rats (Charles River Laboratories, USA; www.criver.com/products-services/find-model/cd-sd-igs-rat?region=3611) and treated for 24 h with IAPP peptides (20 μmol/l) or Tris-HCl buffer (20 mmol/l, pH 7.4) were made available from a previous study [25]. All cDNA samples were analysed in triplicate using pre-validated Taqman gene expression assays (Life Technology, Foster City, CA, USA), with specific probes listed in ESM Table 2. Expression of each gene was calculated using the ΔΔC_t_ method, with *Ppib* or 18S as housekeeping genes.

### Statistical analyses

Data are presented as mean±SEM. Numbers of experimental replications are represented by individual data points in figures. Mean data were compared among treatment groups by one-way ANOVA followed by Holm–Šidák’s multiple comparisons tests. A two-tailed Student *t* test was used when two groups were compared. A *p* value ≤0.05 was considered statistically significant. All statistical analyses were performed using Prism 9 (GraphPad Software, San Diego, CA, USA).

## Results

### Islet ***Plat*** expression and plasmin activity increase under amyloid-forming conditions

**Fig. 1:**
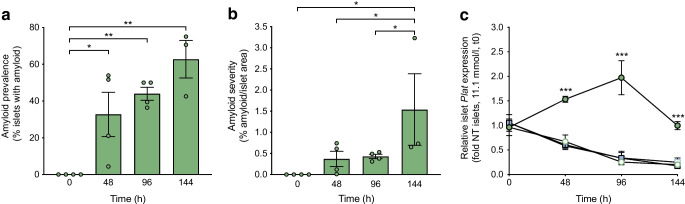
Islet *Plat* expression increases with amyloid formation. (**a**, **b**) Time course of amyloid prevalence (% islets with amyloid; **a**) and severity (% amyloid/islet area; **b**) in hIAPP islets cultured at 16.7 mmol/l glucose. *n*=3–4; *p≤0.05, **p≤0.01. (**c**) Time course of *Plat* mRNA levels in hIAPP transgenic (green circles) and non-transgenic (NT; blue squares) islets cultured for up to 144 h in 11.1 mmol/l (open symbols) or 16.7 mmol/l (closed symbols) glucose conditions. Data are presented as fold expression relative to *Plat* expression in NT islets cultured in 11.1 mmol/l glucose at time 0. *n*=4; ***p≤0.001 for hIAPP islets cultured in 16.7 mmol/l glucose vs hIAPP islets cultured in 11.1 mmol/l glucose, or NT islets cultured in 16.7 mmol/l glucose or 11.1 mmol/l glucose, at the same time point

To validate the RNA-seq data identifying *Plat* as a gene specifically upregulated within islets upon amyloid deposition (2.7 log_2_-fold; *p*=6.76^−56^) [13], we measured its expression in islets from hIAPP transgenic and non-transgenic mice cultured for up to 144 h in 16.7 mmol/l glucose (amyloid-forming conditions) or 11.1 mmol/l glucose (control). As expected, amyloid deposition increased in a time-dependent manner only in hIAPP transgenic islets cultured at 16.7 mmol/l glucose (Fig. 1a,b; data not shown for the 11.1 mmol/l glucose culture condition). Islet *Plat* mRNA levels increased over time in hIAPP transgenic islets under amyloid-forming conditions, whereas this increase was not observed in hIAPP transgenic islets cultured under non-amyloidogenic conditions (11.1 mmol/l glucose) or mIAPP islets cultured in 11.1 mmol/l or 16.7 mmol/l glucose (Fig. 1c). Further, during this 144 h time-course period, *Plat* expression in amyloid-laden hIAPP transgenic islets cultured in 16.7 mmol/l glucose was significantly upregulated by 2.8±0.3-fold (*p*≤0.001), 7.5±2.4-fold (*p*≤0.001) and 4.5±0.6-fold (*p*≤0.001) at 48 h, 96 h and 144 h, respectively, when compared with non-transgenic control islets cultured in 16.7 mmol/l glucose (Fig. 1c).

**Fig. 2:**
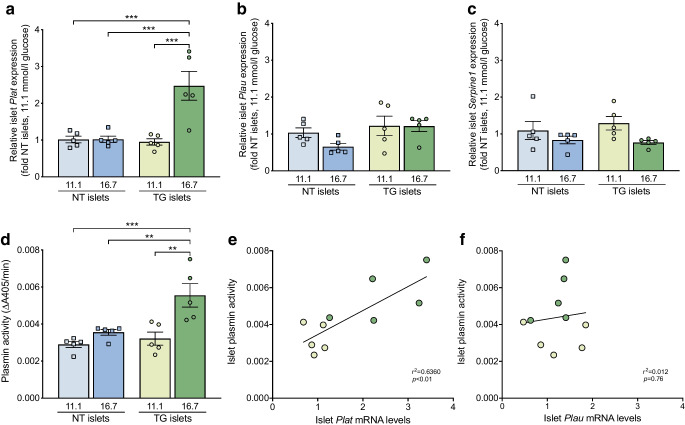
Expression and activity of the components of the fibrinolytic system in islets under amyloid-forming conditions. (**a**–**c**) Quantification of *Plat* (**a**), *Plau* (**b**) and *Serpine1* (**c**) mRNA levels in non-transgenic (NT; blue squares) and hIAPP transgenic (TG; green circles) islets cultured for 48 h in 11.1 mmol/l or 16.7 mmol/l glucose conditions. Data are presented as fold expression relative to expression in NT islets cultured in 11.1 mmol/l glucose. (**d**) Islet plasmin activity in non-transgenic (NT; blue squares) and hIAPP transgenic (TG; green circles) islets cultured for 48 h in 11.1 mmol/l or 16.7 mmol/l glucose conditions. A405, absorbance at 405 nm. (**e**, **f**) Simple linear regression between islet plasmin activity and islet *Plat* (**e**) or *Plau* (**f**) mRNA levels in hIAPP transgenic islets cultured for 48 h in 11.1 mmol/l (light green circles) or 16.7 mmol/l (dark green circles) glucose conditions. *n*=5. **p≤0.01, ***p≤0.001

To determine whether islet expression and activity of other components of the fibrinolytic system were impacted by the process of amyloid deposition, hIAPP transgenic and non-transgenic islets were cultured for 48 h in 11.1 mmol/l or 16.7 mmol/l glucose. *Plat* was upregulated in transgenic islets cultured in 16.7 mmol/l glucose vs transgenic islets cultured in 11.1 mmol/l glucose and non-transgenic islets cultured in 16.7 mmol/l glucose (Fig. 2a). However, there was no change in islet expression of the other fibrinolysis activator, urokinase plasminogen activator (uPA; encoded by *Plau*; Fig. 2b), or plasminogen activator inhibitor-1 (PAI-1; encoded by *Serpine1*; an endogenous inhibitor of tPA and uPA; Fig. 2c). Plasmin activity was significantly increased in transgenic islets under amyloidogenic conditions (Fig. 2d). In hIAPP transgenic islets, plasmin activity was significantly correlated with mRNA levels of *Plat* (Fig. 2e) but not *Plau* (Fig. 2f), suggesting the hIAPP aggregation-induced increase in islet *Plat* expression was associated with increased activity of the fibrinolytic system. Of note, no significant correlation was observed between *Plat* mRNA levels and plasmin activity in non-transgenic islets cultured in 11.1 mmol/l and 16.7 mmol/l glucose (*r*^2^=0.0073, *p*=0.8145), indicating that this was not an effect of glucose per se.

### Islet ***Plat*** expression and plasmin activity are dependent on amyloid formation

**Fig. 3:**
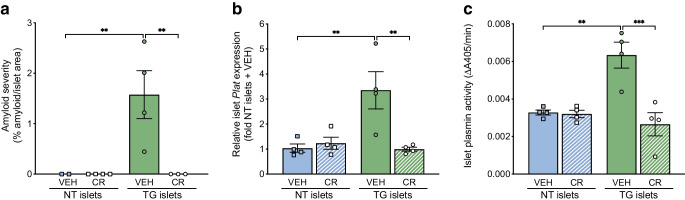
Islet *Plat* expression and plasmin activity are dependent on amyloid formation. (**a**–**c**) Amyloid formation (**a**), *Plat* mRNA levels (presented as fold expression relative to expression in non-transgenic [NT] islets cultured in 16.7 mmol/l glucose with vehicle [VEH]) (**b**) and plasmin activity (**c**) in NT and hIAPP transgenic (TG) islets cultured for 48 h in 16.7 mmol/l glucose conditions in the presence of the known amyloid inhibitor Congo Red (CR; 200 μmol/l) or its VEH (DMSO) control. A405, absorbance at 405 nm. *n*=2–4. **p≤0.01, ***p≤0.001

To evaluate whether the increase in islet *Plat* expression and plasmin activity depended on amyloid formation, hIAPP transgenic and non-transgenic islets were cultured for 48 h in 16.7 mmol/l glucose in the presence or absence of the amyloid inhibitor Congo Red. As expected, amyloid deposition was prevented in hIAPP islets in the presence of Congo Red (Fig. 3a). In parallel, increases in *Plat* expression (Fig. 3b) and plasmin activity (Fig. 3c) in hIAPP transgenic islets were abrogated with Congo Red, while Congo Red had no effect in non-transgenic islets. These findings imply that the increase in *Plat* expression and plasmin activity was downstream of amyloid formation.

### hIAPP increases ***Plat*** expression in macrophages and beta cells but not endothelial cells

**Fig. 4:**
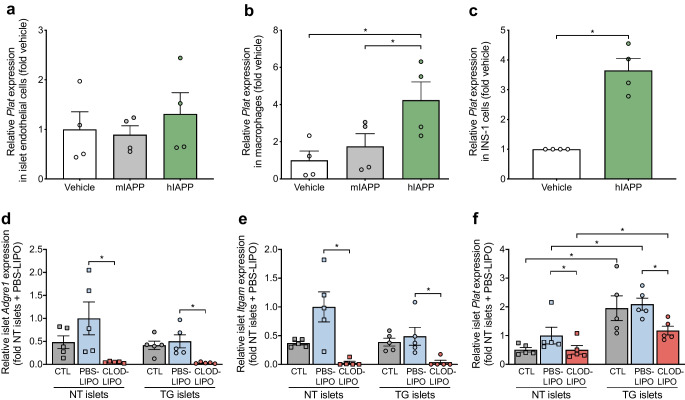
Islet cell types producing tPA in response to hIAPP aggregation. (**a**–**c**) Quantification of *Plat* mRNA levels in primary rat islet endothelial cells (**a**), rat BMDM (**b**) and INS-1 cells (an immortalised beta cell line) (**c**) treated for 24 h with hIAPP (20 µmol/l), or the non-amyloidogenic mIAPP (20 µmol/l) or vehicle (Tris-HCl buffer) as controls. Data are presented as fold expression relative to expression in cells treated with vehicle. *n*=4 independent experiments for each cell type. (**d**–**f**) Quantification of *Adgre1* (**d**), *Itgam* (**e**) and *Plat* (**f**) mRNA levels in hIAPP transgenic (TG) and non-transgenic (NT) islets treated for 48 h with clodronate-containing liposomes (CLOD-LIPO), PBS-containing liposomes (PBS-LIPO) or PBS alone (CTL), and then cultured for 48 h in 16.7 mmol/l glucose conditions. Data are presented as fold expression relative to expression in NT islets treated with PBS-LIPO and then cultured in 16.7 mmol/l glucose. *n*=5. *p≤0.05

We next sought to investigate the islet cell type(s) in which tPA is increased in response to hIAPP aggregation. As endothelial cells are characterised as the major tPA-producing cell type [26] and have been described to be targets of toxic and/or inflammatory effects of amyloid formation [25], we first determined whether hIAPP treatment stimulates *Plat* expression in these cells. Primary rat islet endothelial cells were treated for 24 h with hIAPP, non-amyloidogenic mIAPP, or vehicle, as control. Treatment with 20 μmol/l hIAPP (which we have previously shown to decrease cell viability and increase expression of endothelial cell activation markers [25]) did not increase *Plat* expression in these cells (Fig. 4a).

Since macrophages also produce tPA [27] and can be activated by hIAPP (10 μmol/l) [28, 29], we determined whether hIAPP increases *Plat* expression in these cells. *Plat* mRNA levels were quantified in rat BMDM treated for 24 h with hIAPP, mIAPP or vehicle. *Plat* expression significantly increased by fourfold solely with hIAPP treatment (Fig. 4b). Interestingly, hIAPP was also effective at increasing *Plat* expression in INS-1 beta cells (Fig. 4c), suggesting macrophages may not be the sole source of the hIAPP-induced increase in tPA in the islet.

To further determine the contribution of islet macrophages to tPA upregulation by hIAPP aggregation, isolated hIAPP transgenic and non-transgenic islets were treated with clodronate-containing liposomes to deplete macrophages, or PBS-containing liposomes or PBS alone as controls. Islets were then cultured for 48 h in 16.7 mmol/l glucose to induce amyloid deposition in hIAPP islets, after which islet macrophage markers (*Adgre1* and *Itgam*) and *Plat* mRNA levels were quantified. In both hIAPP transgenic and non-transgenic islets, clodronate treatment significantly abrogated *Adgre1* (Fig. 4d) and *Itgam* (Fig. 4e) expression compared with controls treated with PBS-containing liposome, confirming islet macrophage depletion in both genotypes. As expected, *Plat* expression significantly increased by 2.1-fold in hIAPP islets treated with PBS-containing liposomes vs non-transgenic islets treated the same (Fig. 4f). Clodronate treatment also significantly reduced *Plat* expression (vs islets treated with PBS-containing liposomes) in both hIAPP and non-transgenic islets; however, *Plat* expression was still increased by 2.3-fold in clodronate-containing-liposome-treated hIAPP islets vs non-transgenic islets treated the same (Fig. 4f). These data suggest that, in addition to macrophages, other islet cell types (e.g. beta cells) produce tPA in response to hIAPP aggregation.

### Plasmin protects beta cells from hIAPP-induced cytotoxicity by cleaving hIAPP and inhibiting fibril formation

**Fig. 5:**
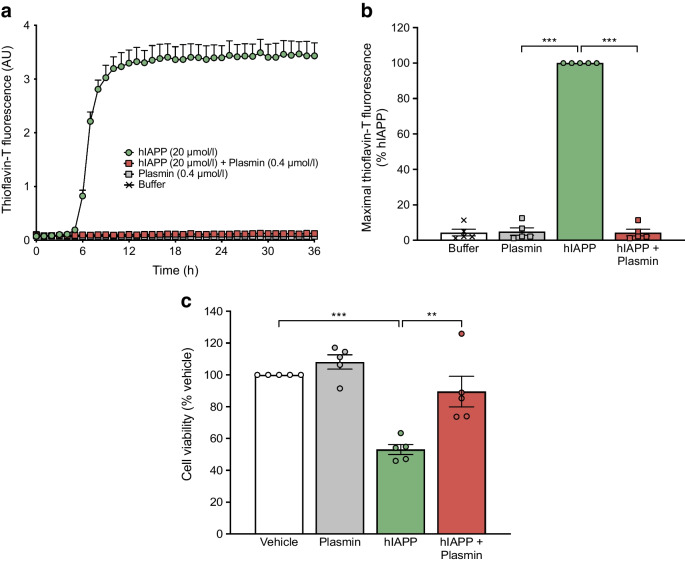
Plasmin reduces hIAPP fibril formation and protects beta cells from hIAPP-induced cytotoxicity. (**a**) Amyloid formation assays of hIAPP (20 μmol/l) carried out in the absence or presence of plasmin (0.4 μmol/l) and monitored for 36 h at 37°C by thioflavin-T fluorescence in a cell-free system. ‘Buffer’ is Tris-HCl (20 mmol/l, pH 7.4). One representative experiment is shown with technical triplicates. AU, arbitrary units. (**b**) Quantification of maximal hIAPP aggregation with hIAPP (20 μmol/l) and/or plasmin (0.4 μmol/l). ‘Buffer’ is Tris-HCl (20 mmol/l, pH 7.4). *n*=5. (**c**) Cell viability (CellTiter-Fluor [CTF] assay) of INS-1 cells treated for 24 h with vehicle (Tris-HCl), hIAPP (20 μmol/l) and/or plasmin (0.4 μmol/l). *n*=5. **p≤0.01, ***p≤0.001

Given that plasmin degrades the amyloidogenic peptide Aβ [18], we next sought to determine whether plasmin also cleaves hIAPP and could, thereby, prevent its aggregation. Using thioflavin-T assays, we found that plasmin decreases hIAPP fibril formation in a dose-dependent manner (ESM Fig. 1), with 0.4 µmol/l plasmin abrogating fibril formation by 95.6% (Fig. 5a,b). In contrast, tPA alone had no measurable effect on hIAPP fibril formation (ESM Fig. 1). Furthermore, treatment of the beta cell line INS-1 for 24 h with hIAPP in the presence or absence of plasmin showed that plasmin prevented hIAPP-induced decreases in cell viability (Fig. 5c).

**Fig. 6:**
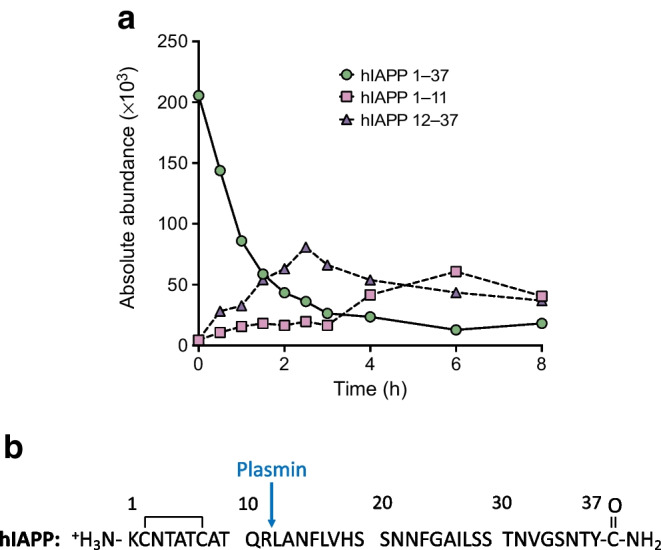
Plasmin cleaves monomeric hIAPP between amino acids 11 and 12. (**a**) Abundance of full-length hIAPP (hIAPP 1–37), hIAPP 1–11 and hIAPP 12–37 measured by LC/MS at 0, 2, 4, 6 and 8 h after combining hIAPP (20 μmol/l) with human plasmin (0.4 μmol/l). (**b**) The primary sequence of hIAPP with the MS-identified site of plasmin cleavage is shown by the blue arrow

Using MS, we found that plasmin cleaves monomeric hIAPP generating hIAPP 1–11 and 12–37 fragments. Incubation of hIAPP with plasmin followed by LC/MS analysis of these cleavage products as a function of time showed that plasmin induces a rapid decrease in the abundance of full-length hIAPP and the appearance of the 1–11 and 12–37 fragments (Fig. 6a), confirming that plasmin cleaves monomeric hIAPP between amino acid 11 and 12 (Fig. 6b).

### hIAPP 12–37 forms amyloid more rapidly than full-length hIAPP, but is not cytotoxic

**Fig. 7:**
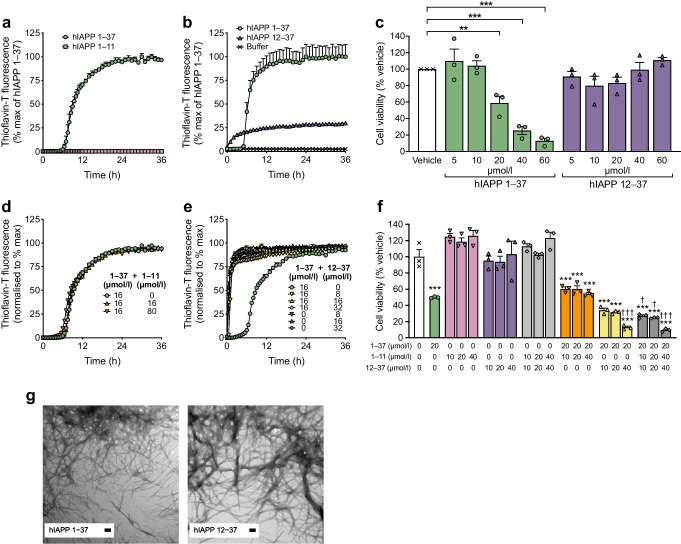
hIAPP 12–37 is not cytotoxic, aggregates faster than hIAPP 1–37 and accelerates amyloid formation by hIAPP 1–37. (**a**, **b**) Thioflavin-T fluorescence profiles of amyloid formation kinetics for full-length hIAPP and the hIAPP 1–11 fragment (**a**), and full-length hIAPP, the hIAPP 12–37 fragment and buffer (Tris-HCl 20 mmol/l, pH 7.4) (**b**), monitored for 36 h at 37°C. In (**a**) and (**b**), one representative experiment is shown with technical triplicates. (**c**) Cell viability (CellTiter-Fluor [CTF] assay) of INS-1 cells treated for 24 h with vehicle or increasing concentrations of hIAPP 1–37 or hIAPP 12–37. *n*=3. **p≤0.01, ***p≤0.001. (**d**, **e**) Thioflavin-T fluorescence profiles of amyloid formation kinetics for full-length hIAPP alone (16 μmol/l) or with the addition of hIAPP 1–11 in ratios of 1:1 and 1:5 (**d**), and full-length hIAPP alone (16 μmol/l) or with the addition of hIAPP 12–37 in ratios of 1:0.5, 1:1 and 1:2, or hIAPP 12–37 alone (**e**), monitored for 36 h at 25°C. In (**d**) and (**e**), one representative experiment is shown with technical triplicates. (**f**) Cell viability (CTF assay) of INS-1 cells treated for 24 h with vehicle (Tris-HCl, 20 mmol/l, pH 7.4), or hIAPP 1–37, 1–11 or 12–37 alone or combined in different ratios. *n*=3. ***p≤0.001 vs vehicle; ^†^*p*≤0.05, ^†††^*p*≤0.001 vs hIAPP 1–37 alone. (**g**) Transmission electron microscopy-derived representative images of full-length hIAPP and hIAPP 12–37. Images were taken at the end of the kinetic reactions. Scale bar, 100 nm

We next assessed the amyloidogenicity and cytotoxicity of hIAPP 1–11 and 12–37. Using thioflavin-T assays, we found that hIAPP 1–11 did not aggregate (Fig. 7a) whereas hIAPP 12–37 aggregated faster than full-length hIAPP (Fig. 7b). While the kinetics of full-length hIAPP aggregation showed a typical initial lag phase followed by a sigmoidal transition to a steady state [30], there was no visible lag phase during aggregation of hIAPP 12–37 (Fig. 7b). Maximal thioflavin-T fluorescence of the fragment was lower than the same concentration of full-length hIAPP (*p*≤0.001; *n*=4). By treating INS-1 cells for 24 h with increasing doses of hIAPP 12–37 or full-length hIAPP, we found that full-length hIAPP reduces cell viability in a dose-dependent manner, while, in contrast, hIAPP 12–37 was not cytotoxic to INS-1 cells, even at 60 µmol/l (Fig. 7c).

Some hIAPP-derived fragments have been shown to inhibit or enhance the aggregation kinetics of full-length hIAPP [11, 31]. Thus, mixtures of full-length hIAPP and 1–11 or 12–37 fragments were analysed to determine whether the fragments modulate amyloid formation by the full-length peptide. The addition of hIAPP 1–11 to hIAPP 1–37 did not alter the aggregation kinetics of full-length hIAPP (Fig. 7d). In concentration ratios of 0.5:1 to 2:1 of 12–37:full-length hIAPP, the mixture aggregated rapidly, indicating that hIAPP 12–37 accelerates aggregation of full-length hIAPP (Fig. 7e). By treating INS-1 cells for 24 h with mixtures of hIAPP 1–37 and the hIAPP fragments, we found that the addition of 1–11 and/or 12–37 fragments to full-length hIAPP did not reverse the 50% reduction in cell viability induced by full-length hIAPP (Fig. 7f). Further, addition of a high concentration (40 μmol/l) of the 12–37 fragment or mixtures of 1–11 and 12–37 fragments to hIAPP 1–37 led to mixtures that were more toxic to INS-1 cells than hIAPP 1–37 alone (Fig. 7f).

Thioflavin-T assays accurately report amyloid formation by wild-type hIAPP and a wide range of IAPP mutants; however, since the dye is an extrinsic probe, it was important to assess amyloid formation with an independent method [32]. Transmission electron microscopy imaging revealed that hIAPP 12–37 fibrils were similar in appearance to those formed by full-length hIAPP (Fig. 7g).

Taken together these data indicate that, by generating hIAPP fragments that alone or when combined with each other are not cytotoxic, plasmin-mediated cleavage of hIAPP may protect beta cells from hIAPP aggregation-induced cytotoxicity.

### Islet ***PLAT*** and ***SERPINE1*** mRNA levels are increased in human type 2 diabetes

**Fig. 8:**
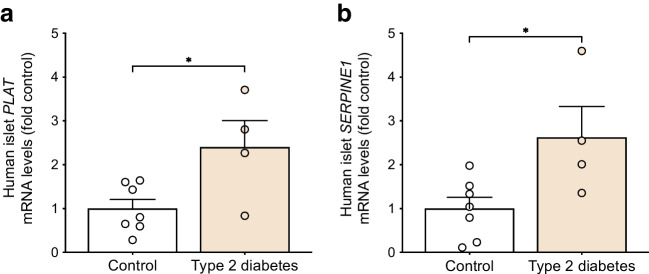
*PLAT* and *SERPINE1* genes are expressed in human islets and increased with type 2 diabetes. (**a**, **b**) Quantification of (**a**) *PLAT* and (**b**) *SERPINE1* mRNA levels in islets from male (M) and female (F) donors with (*n*=4; 1M/3F) or without (*n*=7; 6M/1F) type 2 diabetes. Data are normalised to 18S ribosomal RNA (rRNA) and expressed as fold relative to control. *p≤0.05

To determine whether islet tPA is increased in type 2 diabetes, *PLAT* mRNA levels were measured in islets isolated from donors with and without type 2 diabetes. *PLAT* mRNA levels were increased by 2.4-fold in islets from donors with type 2 diabetes compared with control islets (Fig. 8a). Further, *SERPINE1* mRNA levels were also increased by 2.6-fold in islets from donors with type 2 diabetes compared with control islets (Fig. 8b). Both *PLAT* and *SERPINE1* genes were detectable in islets from male and female donors. Given the sex/gender distribution of human islets received from the organ procurement organisation, it was not possible to do formal analyses of any gender-/sex-based effect.

## Discussion

Islet amyloid deposition, which occurs in the vast majority of people with type 2 diabetes, is associated with beta cell loss and secretory dysfunction, both of which critically contribute to the development of the disease [1–3]. In this study, we delineated a new intra-islet role for the fibrinolytic system in modulating amyloidogenesis. We found islet *Plat* expression and plasmin activity to be specifically upregulated with the aggregation of hIAPP. Further, plasmin cleaved hIAPP, abrogated its aggregation and protected beta cells from hIAPP-induced toxicity. Our data also demonstrated that hIAPP aggregation increases *Plat* expression in islet macrophages and beta cells. Finally, *PLAT* expression was increased in islets from donors with type 2 diabetes.

Apart from its fibrinolytic activity, several studies have reported that the tPA/plasmin system may be involved in amyloidogenesis. Specifically, it has been demonstrated that the tPA/plasmin system is induced by Aβ aggregation in Alzheimer’s disease and can reduce brain amyloid deposition [18–20]. In addition, it has been shown that tPA is able to bind to IAPP fibrils [16]. Moreover, aggregated hIAPP and Aβ can mediate tPA activation of plasminogen in the absence of fibrin [17]. Importantly, our study reinforces a role for the fibrinolytic system in amyloidogenesis by showing that the tPA/plasmin system is upregulated in amyloid-laden islets, and that plasmin reduces hIAPP aggregation and protects beta cells from hIAPP aggregation-induced toxicity. Since plasmin can also be generated from plasminogen through the proteolytic activities of uPA, we measured islet *Plau* expression and found it was not increased in amyloid-laden islets. Further, treatment of hIAPP islets with Congo Red blocked amyloid fibril formation and *Plat* upregulation, suggesting that modulation of *Plat* expression is downstream of amyloid formation. Future work determining the mechanism(s) by which this effect occurs in amyloid-laden islets will be of interest. For example, a potential area for future research may involve the interleukin-1 beta pathway since interleukin-1 beta has been shown to upregulate the tPA/plasmin system in mesangial cells [33], and we and others have demonstrated that hIAPP aggregation increases interleukin-1 beta production in islets [28, 29].

Importantly, we confirmed our findings in human islets, showing that *PLAT* expression is increased with type 2 diabetes. As aforementioned, given the sex/gender distribution of human islets received from the organ procurement organisation, we were unable to formally analyse gender-/sex-based effect. However, gender-/sex-based effects would not be expected given that islet amyloid occurs in both male and female individuals with type 2 diabetes [3]. Further, even though the expression of islet *Serpine1* (encoding PAI-1, the endogenous inhibitor of tPA and uPA) was not affected by amyloid deposition in rodent islets in vitro, we found its expression to be increased in islets from donors with type 2 diabetes. Of note, a previous study has identified PAI-1 as a novel glucose-regulated protein, elevated under high glucose conditions in cultured human islets [34]. These data suggest the tPA/plasmin system could act to reduce islet amyloid accumulation, and the increase in PAI-1 in type 2 diabetes could inhibit a physiological protective effect of the fibrinolytic system in the islet. We also cannot exclude the possibility that the increase in systemic PAI-1 levels and activity in obesity and the metabolic syndrome may have a role in type 2 diabetes development [35–38].

Previous studies have shown that plasmin can degrade peptides other than fibrin, including the amyloidogenic peptide Aβ [18]. In this study we have identified plasmin as an hIAPP-degrading enzyme. Typically, plasmin-targeting cleavage sites are located after the basic amino acids lysine or arginine, but rare cleavages have also been described after histidine or glutamine [39]. Using MS, we found plasmin predominantly cleaves hIAPP at one of the predicted sites, the Arg-11-Leu-12 peptide bond, producing hIAPP 1–11 and 12–37 fragments. This is of importance since we and others have previously reported that several hIAPP-degrading enzymes, such as neprilysin [40, 41], matrix metallopeptidase-9 [11] and insulin-degrading enzyme [42, 43], could be exploited to limit deleterious consequences of amyloid deposition in islets. Of note, toxicity of exogenously added hIAPP or Aβ has been shown to be mediated by toxic oligomers rather than preformed fibrils [30, 44, 45]. While our experimental design did not allow us to separate the effects of the different forms of misfolded hIAPP, the toxic effect of added hIAPP on INS-1 cells was blocked by plasmin, suggesting that the cleavage of monomeric hIAPP by plasmin not only inhibited amyloid formation but, most likely, also inhibited toxic oligomer/protofibril formation. Finally, although we feel this is unlikely, we cannot exclude the possibility that prevention of hIAPP fibril formation by plasmin could be due to binding of hIAPP to plasmin rather than via hIAPP cleavage by plasmin.

We tested the amyloidogenicity and cytotoxicity of plasmin-derived hIAPP fragments. As expected, hIAPP 1–11 did not aggregate and was not toxic to INS-1 cells. When compared with full-length hIAPP, hIAPP 12–37, which contains the proposed critical amyloidogenic region for hIAPP aggregation and cytotoxicity [6], displayed accelerated aggregation kinetics. Further, we also found that the 12–37 fragment accelerates the aggregation of the full-length hIAPP, whereas the 1–11 fragment does not. The more rapid aggregation of hIAPP 12–37 likely results from multiple factors; first, we previously showed that removal of the disulfide accelerates fibril formation in the full-length molecule [46]. Second, absence of the first 11 residues removes two of the charged residues in hIAPP, Lys-1 and Arg-11. The reduction in net charge is expected to accelerate fibril formation [47]. Third, recent cryogenic electron microscopy (cryo-EM)-based models of full-length hIAPP-derived fibrils did not define the first 10–11 residues of the polypeptide within their model(s), suggesting that these residues are not required to form the beta-sheet-rich core of the hIAPP fibrils [48–50]. Caution should be used when interpreting the lower steady-state intensity of hIAPP 12–37 in the thioflavin-T assay. The final thioflavin-T intensities in a set of kinetic assays cannot always be directly related to the amount of amyloid fibrils since they could also be due to weaker binding of the dye, the presence of fewer binding sites or a slight change in the conformation of the bound dye that reduces its fluorescence quantum yield [51]. Importantly and interestingly, irrespective of its biophysical properties, hIAPP 12–37 was not cytotoxic. Our results are in accordance with recent work that studied the effect of plasmin conjugated to quantum dots on hIAPP and reported that the resulting cleavage products are not toxic [52]. The apparent disconnect between the amyloidogenic and cytotoxic effects of hIAPP 12–37 may be explained by the fact that early aggregates of hIAPP are believed to be the major cytotoxic species, whereas fully aggregated hIAPP is inert [30]. During the amyloid fibril formation kinetics, it is well established that the lag phase represents the thermodynamically unfavourable nucleation process whereby individual monomers assemble into oligomeric species [30]. The faster aggregation of hIAPP 12–37, with no evident lag phase when aggregation was monitored using thioflavin-T, likely reduces the duration that beta cells are exposed to oligomeric species and may also lower the steady-state population of oligomers and, therefore, their cytotoxic effects. In contrast, hIAPP 12–37 accelerated aggregation of full-length hIAPP, but this did not protect INS-1 cells from hIAPP toxicity, and even appeared to increase it. In line with this observation, previous studies have reported that the serine-to-glycine substitution at position 20 (S20G) in hIAPP leads to faster aggregation than wild-type hIAPP and renders hIAPP more toxic [53, 54]. The molecular basis of these effects is not understood and these results indicate that there is still a great deal to be learned about the mechanisms by which hIAPP induces toxicity.

The cell type(s) that produce tPA within the islet under amyloid fibril-forming conditions has not been elucidated. This is of particular interest for identifying potential cellular therapeutic targets within the islet that may limit amyloid-induced beta cell toxicity. In the blood, tPA mainly derives from vascular endothelial cells [26]. Studies that have performed single-cell transcriptome profiling of human pancreatic islets have shown that tPA is expressed in human islets, predominantly in endothelial cells [55, 56]. As we recently reported that hIAPP aggregation exerts a cytotoxic and proinflammatory effect on islet endothelial cells [25], we tested whether it could also increase tPA expression in this cell type. Surprisingly, we did not find this to be the case, suggesting endothelial cells do not produce tPA in response to islet amyloid deposition. Other cell types are known to produce tPA, including immune cells [27], neurons [57] and neuroendocrine cells [58, 59]. Of note, tPA has been reported to be in rat islet delta cells [59]. hIAPP aggregation has been shown to stimulate the production of proinflammatory cytokines and chemokines from macrophages [28] and we found that it also increased tPA expression in primary BMDM. However, islet macrophage depletion did not completely prevent hIAPP aggregation-induced tPA upregulation, indicating that macrophages may not be the only cell type in the islet producing tPA under amyloidogenic conditions in vitro. Although our preliminary data suggest that an immortalised beta cell line can produce tPA in response to amyloid formation, more studies are required to confirm these findings.

In summary, we identified for the first time that the fibrinolytic system can be upregulated with hIAPP aggregation in islets and can protect beta cells from hIAPP-induced cytotoxicity. Thus, interventions aimed at increasing islet plasmin activity may reduce or limit hIAPP aggregation and, thereby, improve beta cell survival in type 2 diabetes.

## Supplementary Information

Below is the link to the electronic supplementary material.

ESM (PDF 186 KB)

## Data Availability

The data that support the findings of this study are available from the corresponding author on reasonable request.

## Abbreviations

Aβ: Amyloid beta
BMDM: Bone marrow-derived macrophages
Fmoc: 9-Fluorenylmethylcarbonyl
hIAPP: Human islet amyloid polypeptide
IAPP: Islet amyloid polypeptide
M-CSF: Macrophage colony-stimulating factor
mIAPP: Mouse islet amyloid polypeptide
PAI-1: Plasminogen activator inhibitor-1
TFA: Trifluoroacetic acid
tPA: Tissue plasminogen activator
uPA: Urokinase plasminogen activator
