# Supplementary material for: The islet tissue plasminogen activator/plasmin system is upregulated with human islet amyloid polypeptide aggregation and protects beta cells from aggregation-induced toxicity
Source: Diabetologia. 2024 Sep 9;67(9):1897–911. doi: 10.1007/s00125-024-06161-0 (PMC11410534; doi:10.1007/s00125-024-06161-0)
Supplement: Supplementary file 1 — ESM (PDF 186 KB) [file 125_2024_6161_MOESM1_ESM.pdf]

ELECTRONIC SUPPLEMENTARY MATERIAL

ESM Table 1. Human islet donor characteristics

**Islet preparation**	**1**	**2**	**3**	**4**	**5**	**6**
**MANDATORY INFORMATION**						
Unique identifier	SAMN08769819	SAMN08612554	SAMN08612553	SAMN09228907	SAMN09370567	SAMN09432880
Donor age (years)	45	51	51	62	32	51
Donor sex (M/F)	F	F	M	M	M	M
Donor BMI (kg/m^2)	30.5	32.2	25.5	36.5	28.5	35.6
Donor HbA_{1c} or other measure of blood glucose control	6.6%	5.3%	5.4%	5.8%	5.2%	5.4%
Origin/source of islets	IIDP	IIDP	IIDP	IIDP	IIDP	IIDP
Islet isolation centre	Southern California Islet Cell Resource Center	University of Miami	University of Pennsylvania	Scharp/Lacy Research Institute	University of Wisconsin	Southern California Islet Cell Resource Center
Donor history of diabetes? Please select yes/no from drop down list	Yes	No	No	No	No	No
**If Yes, complete the next two lines if this information is available**						
Diabetes duration (years)	Unknown					
Glucose-lowering therapy at time of death	Unknown					
**RECOMMENDED INFORMATION**						
Donor cause of death						
Warm ischaemia time (h)						
Cold ischaemia time (h)						
Estimated purity (%)						
Estimated viability (%)						
Total culture time (h)[d]						
Glucose-stimulated insulin						

secretion or other functional measurement						
Handpicked to purity? Please select yes/no from drop down list						
Additional notes						

**Islet preparation**	**7**	**8**	**9**	**10**	**11**
**MANDATORY INFORMATION**					
Unique identifier	SAMN09644089	SAMN09844981	SAMN10372788	SAMN11606052	SAMN11791244
Donor age (years)	27	56	50	58	47
Donor sex (M/F)	M	M	F	F	M
Donor BMI (kg/m^2)	32.9	29.3	35.4	34.8	36.1
Donor HbA_{1c} or other measure of blood glucose control	6%	7.6%	9.9%	10.1%	5.7%
Origin/source of islets	IIDP	IIDP	IIDP	IIDP	IIDP
Islet isolation centre	Southern California Islet Cell Resource Center	Southern California Islet Cell Resource Center	Southern California Islet Cell Resource Center	Southern California Islet Cell Resource Center	Southern California Islet Cell Resource Center
Donor history of diabetes? Please select yes/no from drop down list	No	Yes	Yes	Yes	No
**If Yes, complete the next two lines if this information is available**					
Diabetes duration (years)		>10 years	> 10 years	0-5 years	
Glucose-lowering therapy at time of death		Insulin	Insulin	Unknown	
**RECOMMENDED INFORMATION**					
Donor cause of death					

Warm ischaemia time (h)					
Cold ischaemia time (h)					
Estimated purity (%)					
Estimated viability (%)					
Total culture time (h)[d]					
Glucose-stimulated insulin secretion or other functional measurement					
Handpicked to purity? Please select yes/no from drop down list					
Additional notes					

ESM Table 2. TaqMan Probes for gene expression analysis

Gene Name	Species	TaqMan Probe Number
Plat	Mouse	Mm00476931_m1
	Rat	Rn01482578_m1
	Human	Hs00263492_m1
Plau	Mouse	Mm00447054_m1
Serpine1	Mouse	Mm00435858_m1
	Human	Hs00167155_m1
Adgre1	Mouse	Mm00802529_m1
Itgam	Mouse	Mm00434455_m1
Ppib	Mouse	Mm00478295_m1
	Rat	Rn03302274_m1
18S rRNA	Human	HS99999901_s1

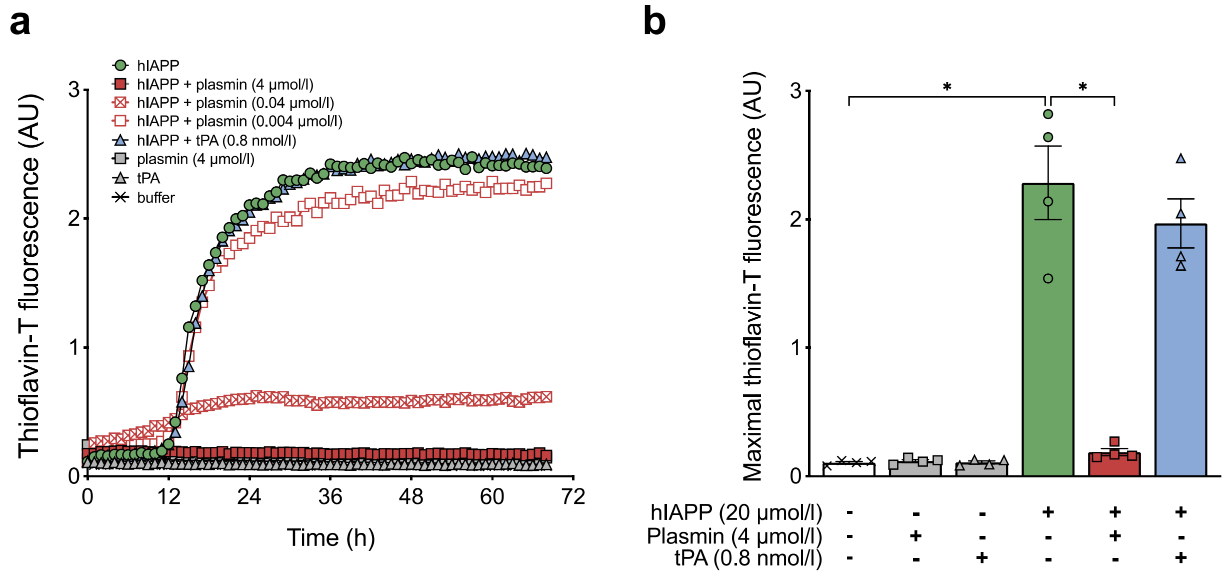

ESM Figure 1. Plasmin, but not tPA, inhibits hIAPP fibril formation in a dose-dependent manner. (a) Representative aggregation of hIAPP (20 µmol/l) carried out in the absence or presence of increasing amounts of plasmin (0.004 to 4 µmol/l) or tPA (0.8 nmol/l) and monitored for 72 hours by thioflavin-T fluorescence in a cell-free system. AU, arbitrary units. (b) Quantification of maximal hIAPP aggregation with or without plasmin or tPA. n=4; *p≤0.05
